# Indocyanine green angiography for lower incidence of anastomotic leakage after transanal total mesorectal excision: a propensity score-matched cohort study

**DOI:** 10.3389/fonc.2023.1134723

**Published:** 2023-06-09

**Authors:** Hengkai Chen, Linfang Ye, Changyu Huang, Yingjun Shi, Fangzhou Lin, Honghao Ye, Yongjian Huang

**Affiliations:** ^1^ Department of Colorectal Surgery, the First Affiliated Hospital, Fujian Medical University, Fuzhou, China; ^2^ Department of Colorectal Surgery, National Regional Medical Center, Binhai Campus of the First Affiliated Hospital, Fujian Medical University, Fuzhou, China; ^3^ Fujian Medical University, Fuzhou, China; ^4^ Fuzhou University, Fuzhou, China; ^5^ Department of Gastrointestinal Surgery 2 Section, the First Affiliated Hospital, Fujian Medical University, Fuzhou, China; ^6^ Department of Gastrointestinal Surgery, National Regional Medical Center, Binhai Campus of the First Affiliated Hospital, Fujian Medical University, Fuzhou, China

**Keywords:** indocyanine green angiography, anastomotic leakage, transanal total mesorectal excision, colonic vascular perfusion, rectal cancer

## Abstract

**Background:**

Anastomotic leakage (AL) is the most serious complication that can arise during colorectal surgery. Indocyanine green (ICG) angiography offers an intraoperative assessment of colonic vascular perfusion in real time. We aimed to assess ICG’s effects on the AL rate in patients who have undergone transanal total mesorectal excision (TaTME) for rectal cancer.

**Methods:**

This retrospective cohort study was conducted at our center from October 2018 to March 2022 to analyze the clinical data of patients with rectal cancer who have undergone TaTME after propensity score matching (PSM). The primary outcome was the proximal colonic transection line modification and clinical AL rate.

**Results:**

A total of 143 patients in the non-ICG group and 143 patients in the ICG group were included after PSM. The proximal colonic transection line of seven patients in the non-ICG group was modified, while 18 were in the ICG group (4.9% *vs.* 12.5%, p = 0.023). Twenty-three patients (16.1%) in the non-ICG group and five patients (3.5%) in the ICG group were diagnosed with AL (p < 0.001). The ICG group had a less hospital readmission rate than the non-ICG group (0.7% *vs.* 7.7%, p = 0.003). The between-group differences in basic line and other outcomes were not significant.

**Conclusions:**

ICG angiography is a safe and feasible method to help surgeons identify potentially poor colonic vascular perfusion and modify the proximal colonic transection line, resulting in a significant reduction in AL and hospital readmission rates.

## Introduction

Anastomotic leakage (AL) is the most severe complication following colorectal surgery with an incidence of 7%–24% (1–4). It can adversely affect both the short- and long-term outcomes, such as the reoperation and hospital admissions rates, along with local recurrence rate and concurrent cancer-specific survival (5–7).

Compared with traditional total mesorectal excision (TME), transanal TME (TaTME), introduced by Lacy et al. (8) in 2010, has several potential benefits in mid/low rectal cancer or difficult cases such as narrow pelvis, bulky tumor, and patients treated with neoadjuvant chemoradiotherapy (CRT), including better specimen quality and radicality, less morbidity and complications, fewer conversions, and more sphincter-preserving rectal resections without compromising oncological outcomes (8–11). In spite of this, the AL rate after TaTME remains high, ranging from 9.8% to 17.9% (12–15). There are three surgery-related factors associated with AL: inadequate anastomosis (16), anastomotic tension (17), and anastomotic vascular perfusion (18–21). In particular, adequate anastomotic vascular perfusion has been emphasized (22–24).

Recently, a real-time and reliable measurement of colonic vascular perfusion can be obtained using near-infrared (NIR) fluorescence imaging with indocyanine green (ICG) (25). It has been demonstrated that ICG angiography might decrease AL rates by selecting a bowel transection site or modifying the transection line according to the demarcation line (26–29). This issue, nevertheless, has been barely studied in TaTME. Further studies are required to verify its efficacy in decreasing the AL rate of patients who underwent TaTME.

We aimed to assess the ICG impact on perioperative outcomes, especially proximal colonic transection line modifying and AL rates in rectal cancer patients treated with TaTME.

## Materials and methods

### Patients and study design

Data from rectal cancer patients who underwent TaTME assisted by laparoscopy between October 2018 and March 2022 at the First Affiliated Hospital of Fujian medical university were used to compile the database. The patients in this study met the following inclusion criteria: 1) malignant tumors were confirmed by computed tomography, magnetic resonance imaging, or pathological diagnosis; 2) clinical records and follow-up information with imaging and physical exam were available. The subsequent exclusion criteria were identified: 1) patients under the age of 18; 2) patients with previous abdominal or pelvic surgery history; 3) patients with multiple primary cancers; 4) patients allergic to ICG or iodine, along with those who were currently receiving iodine dyes or medications likely ICG; 5) conversion to open surgery; 6) emergent cases.

Patients were separated into the ICG and non-ICG groups. Whether patients underwent ICG or clinical assessment evaluation for colonic vascular perfusion was at the discretion of the multidisciplinary team (MDT) and the patient’s intent. The TaTME was performed on each patient with the same surgical group. Patients’ demographics (age, sex, body mass index [BMI], albumin [ALB], comorbidities [including diabetes, hypertension, cardiovascular disease, smoking history, cirrhosis, and steroid use at the time of surgery], and American Society of Anesthesiologists [ASA] scores), tumor features (TNM staging, tumor diameter, distance from the anal verge, and neoadjuvant CRT), operative characteristics (ligation level of inferior mesenteric artery [IMA], anastomosis level from the anal verge, extraction site, operative time, intraoperative blood loss, prophylactic stoma, anastomotic perfusion score, and surgical plan changing including modification of the proximal colonic transection or further surgical operations), and postoperative outcomes [postoperative hospital stay, AL, abdominal/pelvic abscess, surgical reinterventions, ileus, bleeding, acute urinary retention, wound infection and hospital readmission, and other complications with a Clavien–Dindo classification score of grade II or higher occurring during the first 30 days following surgery (30)] were documented in a case report form (CRF).

### Surgical procedure and proximal colon/anastomotic vascular perfusion assessment

TaTME was performed in accordance with previous studies (31). Real-time proximal colonic vascular perfusion assessment was conducted at our center with the laparoscopic NIR camera system provided by Karl Storz (D-Light P; Tuttlingen, Germany) and the Stryker Corporation (1588 AIM Platform, Michigan, USA) just before and after performing the anastomosis by evaluating the mucosa through transanal visualization. Before injecting ICG, the surgeon marked the planned transection colonic line with electrocautery under white light for the initial evaluation. This was performed after the bowel was mobilized, the rectum was transected, the inferior mesenteric vessels were transected, the splenic flexure was mobilized (if it was deemed necessary), and the mesocolon was sectioned, once the specimen had been transabdominally or transanally externalized and before the anastomosis creation. In accordance with the guidance protocol, a bolus of ICG of 0.25 mg/kg was administered intravenously through a peripheral line by the anesthesiology team. The international normalized ratio (INR) was utilized to evaluate colonic perfusion, and the boundary line between the perfused and non-perfused tissue was marked and compared to the planned initial point of the transection. The anastomosis was subsequently created and then another bolus of ICG to evaluate anastomotic perfusion endoluminally (32). The NIR was administered by the transanal device repositioned in the anus. Through the transanal device placed again in the anus, the NIR was introduced. If the surgeon considered that it was required, the patient could receive an ICG third dose (for instance, following an additional surgical procedure including the splenic flexure mobilization if there is too much tension at the mesenteric or anastomotic site, the third injection would be taken).

Proximal colon/anastomotic vascular perfusion was assessed by using an anastomotic perfusion scoring system according to D.A. Sherwinter et al. (33): for clinical assessment in the non-ICG group, dusky appearance was assigned 1 point; patchy appearance was assigned 2 points; pink appearance without pulsatility or bleeding cut edges was assigned 3 points; pink appearance, mesenteric vasculature pulsatility, and bleeding cut edges, but with clinical concern over viability, were assigned 4 points; pink bowel appearance, mesenteric vasculature pulsatility, and bleeding from the cut edge of bowel were assigned 5 points. For fluorescence assessment (30–60 s after ICG injection) in the ICG group, no uptake was marked as 1 point, patchy fluorescence was marked as 2 points, significantly hypofluorescent but homogeneous was marked as 3 points, somewhat hypofluorescent compared to other segments was marked as 4 points, and hypofluorescent to all other segments was marked as 5 points. For both groups, a score of 4 to 5 was considered adequate perfusion for anatomy creation, and 1–2 points indicated poor perfusion, which needed modification of the proximal colonic transection. Whether interventions were needed for patients with 3 points depends on the discretion of MDT and the patient’s condition and intent (32, 33).

### Diagnosis of AL

AL was defined as a defect of the intestinal wall integrity at the anastomosis site (including suture and staple lines of neorectal reservoirs) that permitted connection between intra- and extraluminal compartments regarding the definition and grading of anastomotic leakage of the International Study Group of Rectal Cancer (34), as confirmed by rectal contrast radiologic extravasation evidence or digital rectal examination within 30 days after the operation. According to the impact on clinical management, the severity of AL should be graded. AL of grade A required no modification in the management of patients, AL of grade B required active therapeutic intervention but is manageable without re-laparotomy, and AL of grade C required re-laparotomy (34).

### Statistical analysis

Nearest neighbor propensity score matching (PSM) extracted 1:1 matched pairs of subjects from the non-ICG group or the ICG group based on patient features involving age, sex, BMI, ALB, comorbidities, ASA scores, and tumor features involving tumor diameter, distance from the anal verge, TNM stage, and neoadjuvant CRT. Continuous variables are represented by median (minimum–maximum) or mean ± standard deviations (SDs). To analyze differences in categorical variables, the chi-squared or Fisher’s exact test was applied. The Wilcoxon rank-sum test was utilized to compare continuous variables between groups. p < 0.05 indicated that differences between the two groups were statistically significant. R 3.3.0 was utilized to conduct analyses.

This study was authorized by the Ethics Review Committee of the First Affiliated Hospital of Fujian Medical University [Approval No. (2018)068], and all patients provided written informed permission. All procedures were conducted in conformity with the principles of the Declaration of Helsinki.

## Results

### Patient and tumor characteristics

This study included a total of 370 individuals who underwent TaTME at the First Affiliated Hospital of Fujian medical university. **Table 1** outlines the patient and tumor features that were present before PSM (227 in the non-ICG group and 143 in the ICG group). Before PSM, the ICG group was older and had a shorter distance from the anal verge than the non-ICG group. Following PSM, 143 patients from the non-ICG group and 143 patients from the ICG group were ultimately enrolled in this study. There was no significant difference between the two groups concerning age, sex, BMI, ALB, comorbidities, ASA scores, tumor diameter, distance from the anal verge, TNM stage, and neoadjuvant CRT (**Table 2**).

**Table 1:** Comparison of patient and tumor characteristics before propensity-matched cohort.

Colonic vascular perfusion assessment			
	ICG group n = 143	Non-ICG group n = 227	p-Value
Age (median [range])	69 (41–90) y^a^	64 (22–87) y	<0.001
Sex, M/F	73 (51.0%)/70 (49.0%)	119 (52.4%)/108 (47.6%)	0.798
BMI (median [range])	23.9 (17.0–30.8) kg/m^2^	24.6 (17.4–30.9) kg/m^2^	0.098
ALB (median [range])	41.1 (29.3–53.7) g/L	42.4 (29.1–54.9) g/L	0.078
ASA, I/II/III	8 (5.6%)/128 (89.5%)/7 (4.9%)	17 (7.5%)/194 (85.5%)/16 (7.0%)	0.950
Comorbidities	23 (16.1%)	36 (15.9%)	0.222
Diabetes	10 (7.0%)	18 (7.9%)	0.741
Hypertension	13 (9.1%)	20 (8.8%)	0.928
Cardiovascular disease	6 (4.2%)	11 (4.8%)	0.773
Smoking history	13 (9.1%)	23 (10.1%)	0.743
Cirrhosis	3 (2.1%)	9 (4.0%)	0.325
Steroid use	1 (7.0%)	2 (7.9%)	0.852
Tumor diameter (median [range])	4.3 (1–9) cm	3.9 (1–9) cm	0.066
Distance from the anal verge (median [range])	3.8 (1.6–8.4) cm	4.1 (2.2–9.1) cm	0.034
TNM stage, I/II/III/IV	41 (28.7%)/34 (23.8%)/68 (47.6%)/0 (0%)	50 (22.0%)/66 (29.1%)/108 (47.6%)/3 (1.3%)	0.375
Neoadjuvant CRT^b^	80 (55.9%)	108 (47.6%)	0.118

ICG, indocyanine green; BMI, body mass index; ALB, albumin; ASA, American Society of Anesthesiologists.

a y, years.

b CRT, chemoradiotherapy.

**Table 2:** Comparison of patient and tumor characteristics for propensity-matched cohort.

Colonic vascular perfusion assessment			
	ICG group n = 143	Non-ICG group n = 143	p-Value
Age (median [range])	69 (41–90) y^a^	67 (40–88) y	0.106
Sex, M/F	73 (51.0%)/70 (49.0%)	71 (49.7%)/72 (50.3%)	0.812
BMI (median [range])	23.9 (17.0–30.8) kg/m^2^	24.3 (17.4–30.8) kg/m^2^	0.147
ALB (median [range])	41.1 (29.3–53.7) g/L	42.1 (29.1–54.9) g/L	0.219
ASA, I/II/III	8 (5.6%)/128 (89.5%)/7 (4.9%)	9 (6.3%)/126 (88.1%)/8 (5.6%)	1.000
Comorbidities	23 (16.1%)	28 (19.6%)	0.440
Diabetes	10 (7.0%)	15 (10.5%)	0.297
Hypertension	13 (9.1%)	11 (7.7%)	0.670
Cardiovascular disease	6 (4.2%)	10 (7.0%)	0.303
Smoking history	13 (9.1%)	18 (12.6%)	0.342
Cirrhosis	3 (2.1%)	5 (2.1%)	0.722
Steroid use	1 (7.0%)	1 (7.0%)	1.000
Tumor diameter (median [range])	4.3 (1–9) cm	4.0 (1–9) cm	0.257
Distance from the anal verge (median [range])	3.8 (1.6–8.4) cm	3.9 (2.0–8.3) cm	0.085
TNM stage, I/II/III/IV	41 (28.7%)/34 (23.8%)/68 (47.6%)/0 (0%)	38 (26.6%)/28 (19.6%)/71 (49.7%)/2 (1.4%)	0.500
Neoadjuvant CRT^b^	80 (55.9%)	75 (52.4%)	0.553

ICG, indocyanine green; BMI, body mass index; ALB, albumin; ASA, American Society of Anesthesiologists.

a y, years.

b CRT, chemoradiotherapy.

### Operative characteristics

Operative details are presented in **Table 3**. In terms of operative features, there was no statistically significant difference between groups including ligation level of IMA, anastomosis level from the anal verge, extraction site, operative time, intraoperative blood loss, and prophylactic stoma. Eighteen patients underwent surgical plan changes according to ICG evaluation and seven patients according to clinical evaluation (12.5% *vs.* 4.9%, p = 0.023).

**Table 3:** Operative characteristics.

Colonic vascular perfusion assessment			
	ICG group n = 143	Non-ICG group n = 143	p-Value
Operative time (mean ± SD^a^)	178 ± 36 min^b^	171 ± 34 min	0.451
Blood loss (median [range])	200 (50–450) ml	200 (50–450) ml	0.911
Extraction site, transanal/transabdominal	129 (90.2%)/14 (9.8%)	125 (87.4%)/18 (12.6%)	0.453
Ligation level of IMA^c^, high/low ligation	139 (97.2%)/4 (2.8%)	134 (93.7%)/9 (6.3%)	0.156
Anastomosis level from the anal verge	2.3 ± 1.2 cm	2.6 ± 1.1 cm	0.080
Prophylactic stoma	122 (85.3%)	124 (86.7%)	0.733
Anastomotic perfusion score, 1/2/3/4/5	0 (0%)/2 (1.4%)/16 (11.2%)/27 (18.9%)/98 (68.5%)	0 (0%)/2 (1.4%)/5 (3.5%)/33 (23.1%)/103 (72.0%)	0.180
Change in surgical plan	18 (12.5%)	7 (4.9%)	0.023

ICG, indocyanine green.

a SD, standard deviation.

b min, minutes.

c IMA, inferior mesenteric artery.

### Postoperative outcomes

A total of AL was observed in 28 patients: 5 in the ICG group and 23 in the non-ICG group (3.5% *vs.* 16.1%, p < 0.001, **Table 4**). Among them, 4 (2.8%), 1 (0.7%), and 0 (0%) in the ICG group and 12 (8.4%), 11 (7.0%), and 1 (0.7%) in the non-ICG group were diagnosed with AL of grade A, B, or C (p = 0.040, 0.006, and 1.000, respectively), while 80 (55.9%) in the ICG group and 75 (52.4%) in the non-ICG group underwent neoadjuvant CRT. The ICG group had a less hospital readmission rate than the non-ICG group (0.7% *vs.* 7.7%, p = 0.003, **Table 4**). Abdominal/pelvic abscess, surgical reinterventions, ileus, bleeding, acute urinary retention, wound infection, postoperative hospital stay, and other complications with a Clavien–Dindo classification score of grade II or higher were similar between the two groups (**Table 4**). In addition, surgical reintervention with a stoma was required in one patient diagnosed with AL of grade C in the non-ICG group, while the other 27 patients diagnosed with AL of grade A or B were treated conservatively with antibiotics and CT scan drainage (not shown in **Table 4**).

**Table 4:** Postoperative complications.

Colonic vascular perfusion assessment			
	ICG group n = 143	Non-ICG group n = 143	p-Value
Postoperative hospital stay (median [range])	10 (7–18) d^a^	10 (8–19) d	0.243
AL	5 (3.5%)	23 (16.1%)	<0.001
Grade A	4 (2.8%)	12 (8.4%)	0.040
Grade B	1 (0.7%)	10 (7.0%)	0.006
Grade C	0 (0.0%)	1 (0.7%)	1.000
Abdominal/pelvic abscess	1 (0.7%)	3 (2.1%)	0.622
Ileus	2 (1.4%)	2 (1.4%)	1.000
Bleeding	1 (0.7%)	1 (0.7%)	1.000
Acute urinary retention	1 (0.7%)	2 (1.4%)	1.000
Wound infection	2 (1.4%)	3 (2.1%)	1.000
Hospital readmission	1 (0.7%)	11 (7.7%)	0.003
Surgical reintervention	0 (0%)	1 (0.7%)	1.000
Other complications^b^	3 (2.1%)	4 (2.8%)	1.000

ICG, indocyanine green; AL, anastomotic leakage.

a d, days.

b According to Clavien–Dindo Class score ≥grade II.

Typical cases: Clinical outcome of transection line modification or not according to anastomotic perfusion score evaluated by ICG or clinical assessment.

Case 1 in the ICG group: male, 75 years old, BMI was 26.5 kg/m^2^, tumor diameter was 4.3 cm, TNM stage was IIIB, underwent neoadjuvant CRT, distance from the anal verge was 3.7 cm, anastomotic perfusion score was 2 points, transection line was modified to the level of excellent perfusion, and an anastomosis was created using the modified transection line, with a prophylactic stoma. Clinical outcome: no AL.

Case 2 in the non-ICG group: male, 79 years old, BMI was 27.8 kg/m^2^, with hepatitis B cirrhosis, tumor diameter was 5.1 cm, TNM stage was IIIB, underwent neoadjuvant CRT, distance from the anal verge was 4.2 cm, anastomotic perfusion score was 3 points, anastomosis was carried out with the planned transection line, with a prophylactic stoma. Clinical outcome: AL.

## Discussion

AL is among the most severe postoperative complications following colorectal surgery (35, 36). The most crucial intraoperative factor of AL is anastomotic perfusion (37, 38). ICG angiography is a practical and repeatable method that permits real-time monitoring of tissue perfusion, which aids the surgeon in the visualization of the proximal colonic transection line. The effectiveness of intraoperative ICG angiography in reducing AL rate after colorectal surgery is reported in many studies (15, 39, 40), while few studies were focused on its application in patients who underwent TaTME. This is the first PSM retrospective cohort study to assess ICG angiography’s impact on the incidence of AL in patients who underwent TaTME.

We found that the basic line of patient and tumor characteristics between the two groups did not differ between the two groups following PSM. For operative details, 12.5% of patients in the ICG group underwent surgical plan changes according to ICG evaluation while 4.9% of patients in the non-ICG group according to clinical evaluation (p = 0.023). As a result of that, 5 AL was observed in the ICG group, while 23 in the non-ICG group (3.5% *vs.* 16.1%, p < 0.001) and ICG group had less rate of hospital readmission (0.7% *vs.* 7.7%, p = 0.003). The between-group differences in other operative outcomes were not significant.

Since then, methods including bleeding, palpable pulse in the mesocolon, and intestinal coloration have been employed to evaluate tissue perfusion. Nevertheless, these evaluations are reliant on the surgeon’s clinical judgment, which underestimates the AL risk (41, 42). These results were verified by Jafari et al. (43), who found that the use of who Firefly system led to a 19% change in the proximal resection margin, as opposed to a 4.5% change by the clinical evaluation during low anterior robotic resections, hence reducing the AL rate by 60%–65%. Based on the studies discussed above, conventional methods are not entirely reliable for evaluating bowel perfusion (42, 44). Kim et al. (43) evaluated ICG angiography’s impact on AL rate in patients receiving anterior robotic resections and reported an overall decrease of 4.6% (ICG group 0.8% *vs.* control group: 5.4%, p = 0.03). Kin et al. (45) observed that ICG angiography revealed a shift in the proximal colonic transection in eight patients (5%), and one of them was diagnosed with AL. However, the between-group difference in the AL rate was not significant. Kawada et al. (46) reported that the usage of ICG altered the proximal colonic transection line in 30.9% of the patients undergoing laparoscopic left hemicolectomy. As a result, three patients with a change in the transection line were diagnosed with AL. Mizrahi et al. recently showed that ICG led to a transection line modification in four patients (13.3%), and none of these four patients experienced AL (47). The PILLAR II multicenter study (28), the prospective study with the largest published cases to date, included 139 patients who had ICG evaluation during left hemicolectomy. The surgical plan was changed in 11 patients (7.9%) according to ICG evaluation, none of whom had AL. The AL rate diagnosed in our center (16.1% in the control group) was comparable to that in previous studies (48, 49). Applications for ICG angiography led to modifying the proximal colonic transection line in 12.5% of patients. Typically, in case 1, after proximal colon dissection, the anastomotic perfusion score was 2 points according to ICG evaluation. Therefore, the proximal colonic transection line was modified, and re-evaluation was performed (the score was 5 points). No AL occurred within 30 days of follow-up. Notably, in case 2, after proximal colon dissection, the anastomotic perfusion of the patient was 3 points. Considering his comorbidity of hepatitis B cirrhosis affecting anesthetics-metabolism function of the liver resulting in prolongation of emergence time from anesthesia or postoperative delirium, the proximal colonic transection line was not modified according to the intraoperative discretion of MDT and the patient’s family’s intent. AL (grade 2) occurred on day 7 after the operation. These results indicated that there was indeed great potential for modifying surgical plans according to ICG angiography to decrease the AL rate in patients undergoing TaTME.

Even though we performed many such procedures to minimize potential bias, there is still some room for further improvement. First, this study was based on retrospective data and, thus, there were inevitably some inherent limitations including various biases, such as selection bias. In the future, larger, multi-institutional, prospective, randomized controlled trials are required to validate the efficacy of ICG in preventing AL in patients having TaTME. Second, more cases of patients could show its further strengths in preventing AL. Finally, the evaluation of the intensity of the ICG fluorescence is a subjective process, and through visual assessment for ICG fluorescence, surgeons sometimes have difficulty determining whether or not intestinal perfusion is adequate despite the fact that we have restrictedly qualified the amount of time available for fluorescence evaluation (30–60 s after ICG injection). While there were already some studies with quantitative evaluations in colorectal surgery (40, 46, 48), whether the outcomes may be enhanced further will be revealed by additional research.

In conclusion, this cohort study is the first one to investigate the effect of ICG angiography in decreasing AL rate during TaTME using a PSM analysis. Our results showed that compared to clinical evaluation, ICG angiography, with safety and feasibility, could help surgeons to identify potentially poor colonic vascular perfusion and modify the proximal resection line in a considerable number of patients during TaTME, significantly reducing the AL and hospital readmission rate.

## Data availability statement

The original contributions presented in the study are included in the article/Supplementary Material. Further inquiries can be directed to the corresponding author.

## Ethics statement

The studies involving human participants were reviewed and approved by Ethics Review Committee of the First Affiliated Hospital of Fujian Medical University (Approval No. (2018) 068). The patients/participants provided their written informed consent to participate in this study.

## Author contributions

YH, HC, LY, and CH designed this work; LY, YS, HY, and FL collected, analysis and interpreted data; HC, LY, and CH drafted and review the manuscript; YH critically revised the manuscript and overall supervision. All authors contributed to the article and approved the submitted version.
